# Collagen Induces a More Proliferative, Migratory and Chemoresistant Phenotype in Head and Neck Cancer via DDR1

**DOI:** 10.3390/cancers11111766

**Published:** 2019-11-09

**Authors:** Sook Ling Lai, May Leng Tan, Robert J. Hollows, Max Robinson, Maha Ibrahim, Sandra Margielewska, E. Kenneth Parkinson, Anand Ramanathan, Rosnah Binti Zain, Hisham Mehanna, Rachel J. Spruce, Wenbin Wei, Ivy Chung, Paul G. Murray, Lee Fah Yap, Ian C. Paterson

**Affiliations:** 1 Department of Oral and Craniofacial Sciences, Faculty of Dentistry, University of Malaya, Kuala Lumpur 50603, Malaysia; sl0318ling@gmail.com (S.L.L.); mayleng4@gmail.com (M.L.T.); yapleefah@um.edu.my (L.F.Y.); 2 Institute of Immunology and Immunotherapy, University of Birmingham, Birmingham B15 2TT, UK; R.J.Hollows@bham.ac.uk (R.J.H.); ibrahim.maha@gmail.com (M.I.); S.Margielewska@bham.ac.uk (S.M.); wenbin.wei2@durham.ac.uk (W.W.); p.g.murray@bham.ac.uk (P.G.M.); 3 Centre for Oral Health Research, Newcastle University, Newcastle NE2 4BW, UK; max.robinson@newcastle.ac.uk; 4 South Egypt Cancer Institute, Assiut University, Assiut 71515, Egypt; 5 Centre for Immunobiology and Regenerative Medicine, Institute of Dentistry, Barts and the London School of Medicine and Dentistry, Queen Mary University of London, London E1 4NS, UK; e.k.parkinson@qmul.ac.uk; 6 Department of Oral and Maxillofacial Clinical Sciences, Faculty of Dentistry, University of Malaya, Kuala Lumpur 50603, Malaysia; drranand@um.edu.my; 7 Oral Cancer Research & Coordinating Centre, Faculty of Dentistry, University of Malaya, Kuala Lumpur 50603, Malaysia; profrosnah@mahsa.edu.my; 8 Faculty of Dentistry, MAHSA University, Bandar Saujana Putra 42610, Malaysia; 9 Institute of Head and Neck Studies and Education (InHANSE), University of Birmingham, Birmingham B15 2TT, UK; h.mehanna@bham.ac.uk (H.M.); r.spruce@bham.ac.uk (R.J.S.); 10 Institute of Cancer and Genomic Sciences, University of Birmingham, Birmingham B15 2TT, UK; 11 Department of Biosciences, Durham University, Durham DH1 3LE, UK; 12 Department of Pharmacology, Faculty of Medicine, University of Malaya, Kuala Lumpur 50603, Malaysia; ivychung@ummc.edu.my; 13 Health Research Institute, University of Limerick, Limerick V94 T9PX, Ireland

**Keywords:** head and neck cancer, collagen, DDR1

## Abstract

Head and neck squamous cell carcinoma (HNSCC) is the sixth most common cancer worldwide and includes squamous cell carcinomas of the oropharynx and oral cavity. Patient prognosis has remained poor for decades and molecular targeted therapies are not in routine use. Here we showed that the overall expression of collagen subunit genes was higher in cancer-associated fibroblasts (CAFs) than normal fibroblasts. Focusing on collagen8A1 and collagen11A1, we showed that collagen is produced by both CAFs and tumour cells, indicating that HNSCCs are collagen-rich environments. We then focused on discoidin domain receptor 1 (DDR1), a collagen-activated receptor tyrosine kinase, and showed that it is over-expressed in HNSCC tissues. Further, we demonstrated that collagen promoted the proliferation and migration of HNSCC cells and attenuated the apoptotic response to cisplatin. Knockdown of DDR1 in HNSCC cells demonstrated that these tumour-promoting effects of collagen are mediated by DDR1. Our data suggest that specific inhibitors of DDR1 might provide novel therapeutic opportunities to treat HNSCC.

## 1. Introduction

Head and neck squamous cell carcinoma (HNSCC) is the sixth most common cancer worldwide and includes squamous cell carcinomas of the oropharynx and oral cavity (OPSCC and OSCC, respectively) [1,2]. HNSCC is caused primarily by tobacco and alcohol usage, but a significant proportion of OPSCCs in Western countries are associated with human papillomavirus (HPV) infection [3]. Although patients with HPV-related disease respond better to chemo-radiotherapy and have a more favourable prognosis, approximately 50% of patients with HPV-negative HNSCC die within 5 years. Molecular targeted therapies are not in routine use and innovations in the therapeutic approach are urgently needed. 

The molecular basis of HNSCC has been intensively investigated and a number of mutations in oncogenes (e.g., PIK3CA, RAS) and tumour suppressor genes (e.g., p16, p53, FAT1, NOTCH1) have been identified in subsets of tumours [4], but the identification of druggable targets has proved to be challenging. Like all solid tumours, HNSCCs are complicated structures representing interactions between the malignant tumour cells with normal cells and extracellular matrix (ECM) proteins within the tumour microenvironment. Cancer-associated fibroblasts (CAFs) are often the most abundant cells in the HNSCC microenvironment and serve to promote tumour progression by secreting cytokines/growth factors and ECM proteins [5]. Importantly, high levels of CAFs within OSCCs predict a worse patient prognosis [6], which suggests that therapeutic strategies to inhibit CAF function might be beneficial.

Discoidin domain receptor 1 (DDR1) is a receptor tyrosine kinase that specifically binds to and is activated by collagen [7]. DDR1 binding to collagen occurs through the discoidin domains of DDR1 and is integrin independent. Following activation by collagen, DDR1 triggers the activation of a number of downstream signalling pathways [8], which induces the expression of pro-inflammatory mediators as well as matrix degrading enzymes. The overexpression of DDR1 has been reported in a number of cancer types [8], including breast and lung cancers, and is often associated with more migratory and invasive phenotypes [9,10]. Preliminary evidence from micro-array analysis indicated that DDR1 was over-expressed in SCCs of the tongue [11], but further studies to confirm mRNA or protein expression in HNSCC have not been reported and the role of DDR1 in the pathogenesis of HNSCC has not been examined. 

Here we showed that HNSCCs are collagen-rich environments, with individual collagen subtypes being expressed by both CAFs and malignant epithelial cells. Further, we demonstrated that DDR1 is over-expressed in HNSCC tissues and that collagen promotes the proliferation and migration of HNSCC cells and attenuates the apoptotic response to cisplatin via DDR1. Specific inhibitors of DDR1, therefore, might provide novel therapeutic opportunities to treat HNSCC. 

## 2. Results

### 2.1. Collagen Expression in CAFs and HNSCCs

**Figure 1:**
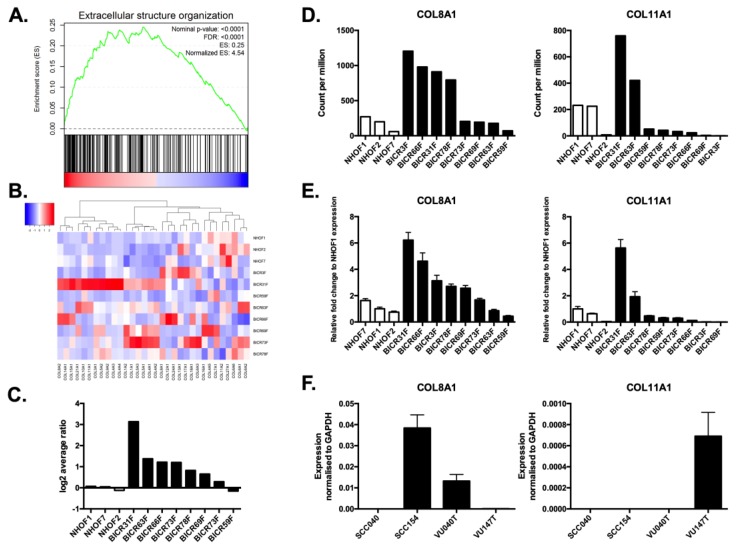
Collagen expression in cancer-associated fibroblasts (CAFs) and head and neck squamous cell carcinoma (HNSCC) cells. (**A**) Extracellular structure organization was the most significantly enriched gene ontology (GO) term identified by gene set enrichment analysis (GSEA) of genes differentially expressed between normal human oral fibroblasts (NHOFs) and CAFs, as determined by RNAseq. (**B**) Heatmap highlighting the elevated expression of collagen subtypes in CAFs. (**C**) Total collagen expression (average expression of all collagen subtypes from RNAseq) was higher in the majority of CAF strains compared to normal fibroblasts. (**D**) Expression of COL8A1 and COL11A1 in NHOFs and CAFs, as determined by RNAseq. (**E**) RT-qPCR was used to confirm the expression of COL8A1 and COL11A1 in fibroblast strains. (**F**) RT-qPCR analysis showed COL8A1 and COL11A1 were also expressed in some HNSCC cell lines. Data for the RT-qPCR experiments are shown as mean +/− SD values of triplicates.

To identify pathways relevant to the pro-tumorigenic effects of CAFs in HNSCC, we used RNA sequencing (RNAseq) to compare global gene expression in a panel of CAFs derived from HNSCCs with that in a panel of normal human oral fibroblasts (NHOF). Gene set enrichment analysis (GSEA) of genes differentially expressed between NHOFs and CAFs revealed that the most significantly enriched gene ontology (GO) term was extracellular structure organization (Figure 1A). As the genes encoding the individual collagen subtypes represented approximately 10% of the genes in this GO term, we examined the expression of individual collagen subtypes in more detail. Whilst there was heterogeneity in the expression of individual collagen subunit genes, we observed that the overall expression of collagen subunit genes was higher in CAFs compared to NHOFs (Figure 1B–D, Figure S1). 

**Figure 2:**
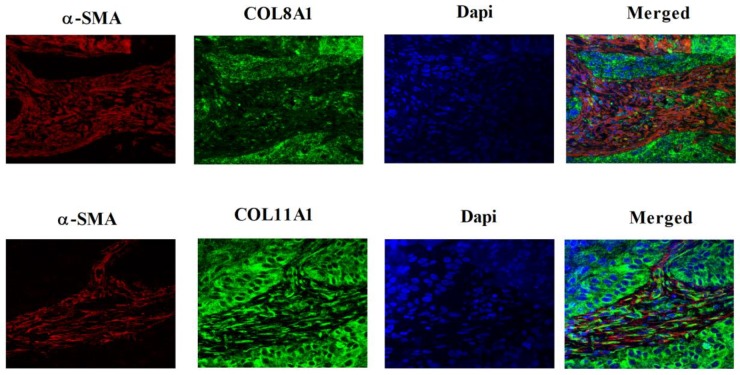
Expression of COL8A1 and COL11A1 in oropharyngeal squamous cell carcinoma (OPSCC). Photomicrographs show expression of COL8A1 (top panel) and COL11A1 (lower panel) in OPSCC tissues. Tissues were multiplex-stained with COL8A1 or COL11A1 (fluorescein, green), together with α-smooth muscle actin (α-SMA) (Cy3, red) antibodies and 4′,6-diamidino-2-phenylindole (DAPI) (blue). The expression of COL8A1and COL11A1 was detected in OPSCCs and cancer-associated fibroblasts. Representative images are shown and were captured with a confocal laser microscope (Zeiss, Carl Zeiss AG, Oberkochen, Germany; magnification × 63). Examples of COL8A1and COL11A1 expression in oral squamous cell carcinoma tissues are shown in Supplementary Figure S2.

As COL8A1 and COL11A1 had previously been shown to also be up-regulated in senescent OSCC-derived CAFs [12], we selected these collagen subtypes for further investigation and their expression in fibroblast strains was first validated by RT-qPCR (Figure 1E). The expression of COL8A1 and COL11A1 was also detected in HNSCC-derived cell lines, although expression levels were heterogeneous and unrelated to HPV status (Figure 1F). We next examined whether COL8A1 and COL11A1 were expressed in HNSCC tissues. A series of OPSCC tissue samples were dual-stained for COL8A1 or COL11A1, together with alpha-smooth muscle actin (α-SMA), which is a marker of fibroblast activation. Both COL8A1 and COL11A1 were found to be expressed by OPSCC cells and CAFs (Figure 2). Specifically, 73% (32/44) of OPSCCs and 91% of the associated CAFs (40/44) expressed detectable levels of COL8A1, and 100% of OPSCCs (55/55) and 98% of the associated CAFs (54/55) expressed COL11A1. Similarly, both collagens were expressed by tumour cells (100% of cases) and CAFs (97% of cases) in OSCCs (Figure S2). 

The expression of COL8A1 or COL11A1 was examined in the context of clinico-pathological parameters. Low expression of COL8A1 in OPSCCs (*p* = 0.004) and CAFs (*p* = 0.048) was significantly associated with the low-risk-of-death group by univariate logistic regression (Table S1). Survival analyses indicated that high expression of COL8A1 in OPSCCs and CAFs was associated with worse survival, but this was not statistically significant under Kaplan–Meier analyses (data not shown). The expression of COL11A1 was not associated with any clinico-pathological parameters and no associations were found for either COL8A1 or COL11A1 in OSCCs. 

### 2.2. DDR1 Is Over-Expressed in HNSCCs 

**Figure 3:**
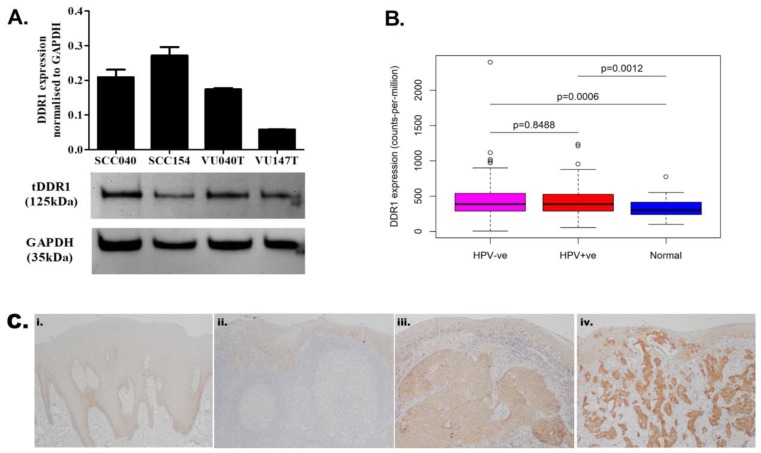
Discoidin domain receptor 1 (DDR1) was over-expressed in head and neck squamous cell carcinoma (HNSCC). (**A**) DDR1 is readily detectable in HNSCC cell lines by RT-qPCR and western blotting. (**B**) Analysis of The Cancer Genome Atlas (TCGA) expression data revealed that DDR1 is significantly over-expressed in tumours relative to normal samples. There was no statistically significant difference in DDR1 expression between human papillomavirus (HPV)-negative and HPV-positive tumours. (**C**) Immunohistochemical analysis of DDR1 protein revealed that normal epithelium showed weak cytoplasmic staining (i and ii), whilst the majority of squamous cell carcinomas (8 of 12) showed increased DDR1 expression in comparison to normal epithelium (iii and iv). (Original magnification × 100).

Having demonstrated collagen expression in both tumour cells and CAFs, we next examined the expression of DDR1, a collagen-activated tyrosine kinase receptor. DDR1 mRNA and protein were readily detected in HNSCC cell lines (Figure 3A, Figure S3) and the data indicated that the expression of DDR1 was higher in HNSCC cell lines than immortalized normal human oral keratinocytes and non-malignant epidermal keratinocytes (Figure S4). To investigate DDR1 expression in HNSCC tissues, we first used expression data from The Cancer Genome Atlas (TCGA). DDR1 was significantly over-expressed in tumours relative to normal samples, and this was the case for both HPV-negative (*p* = 0.0006) and HPV-positive tumours (*p* = 0.0012; Figure 3B). To confirm these data at the protein level, we first used immunohistochemistry to examine the expression of DDR1 in a small series of cases comprising 5 cases of normal oral mucosa, 6 cases of OPSCC and 6 cases of OSCC (Figure 3C). Normal epithelium showed weak cytoplasmic staining, whilst the majority of squamous cell carcinomas (8 of 12) showed increased DDR1 expression in comparison to adjacent normal epithelium (Table S2).

**Figure 4:**
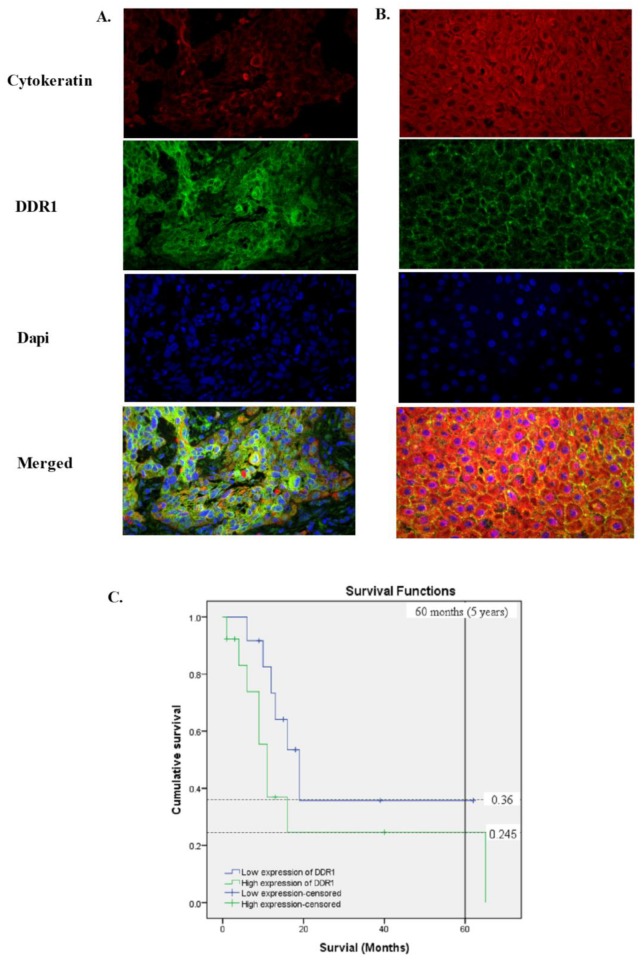
Expression of discoidin domain receptor 1 (DDR1) in oropharyngeal squamous cell carcinoma (OPSCC). Tissues were multiplex-stained with pan-cytokeratin cocktail AE1/AE3 (Cy3, red) and DDR1 (fluorescein, green) antibodies, plus 4′,6-diamidino-2-phenylindole (DAPI) (blue) nuclear counterstain. DDR1 expression in OPSCCs was (**A**) cytoplasmic and membraneous or (**B**) membraneous. Representative images are shown and were captured using Metamorph Pathology Imaging System (Nikon, Tokyo, Japan; magnification ×60). Examples of DDR1 expression in oral squamous cell carcinoma tissues are shown in Supplementary Figure S5. (**C**) High DDR1 expression in OPSCC patients was correlated with worse survival. Patients with high DDR1 expression have a lower 5-year survival rate (33%) than that of patients with low DDR1 expression (78%), log-rank (Mantel–Cox) (*p* = 0.022).

We next examined the tissue and subcellular localisation of DDR1 in more detail using multiplex immunofluorescence staining of formalin-fixed paraffin-embedded tissue sections. Pan-cytokeratin was used to highlight the epithelium. DDR1 expression was localised to the malignant keratinocytes and was detected in the majority of OPSCCs (95%, 53/56) of OPSCC tissues examined and the staining was cytoplasmic and membraneous or predominantly membraneous (Figure 4A,B). The staining pattern was similar in OSCCs (Figure S5) and DDR1 was expressed in 97% (41/42) of OSCCs examined. 

For OPSCCs, univariate logistic regression analyses indicated that low DDR1 expression was significantly associated with the low-risk-of-death group (*p* = 0.036; Table S1). In support of these data, Kaplan–Meier survival analysis demonstrated that, in this small cohort (53 OPSCC cases with survival data), patients with high DDR1 expression had a significantly worse survival outcome (*p* = 0.022) compared to cases showing low expression (Figure 4C). Survival data were available for only 25 OSCC cases, so meaningful comparisons were not possible. 

### 2.3. Collagen Stimulates Proliferation and Migration and Suppresses the Response of HNSCC Cells to Cisplatin

**Figure 5:**
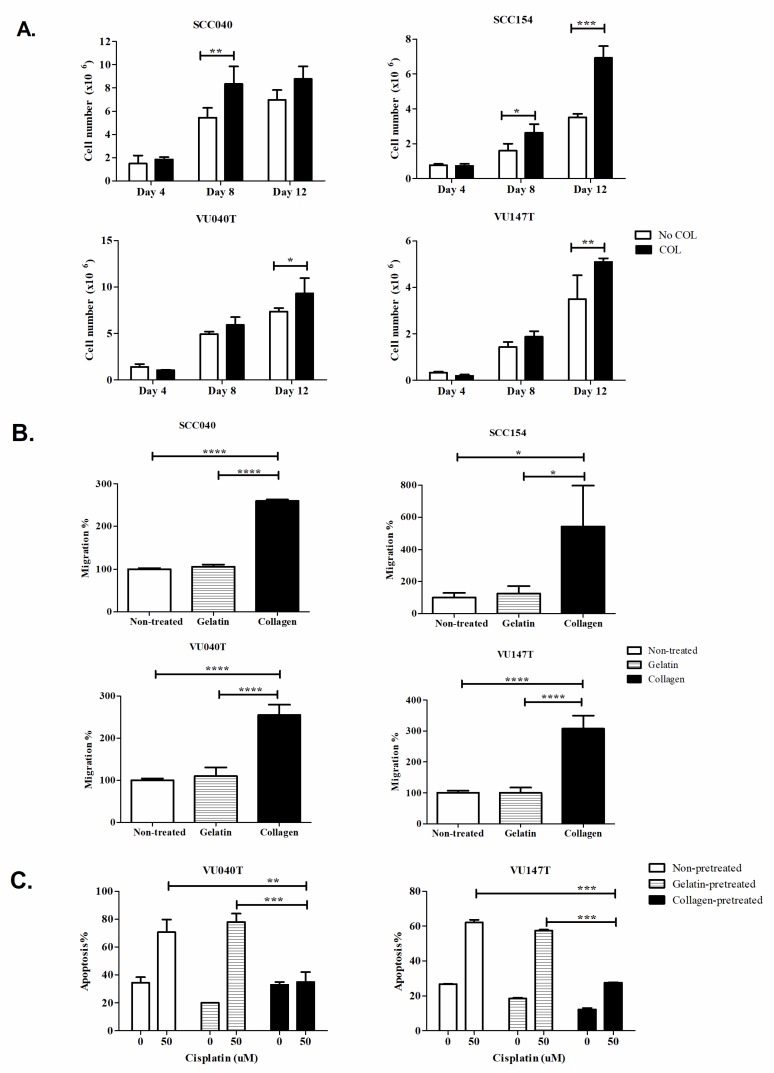
Collagen promoted the proliferation and migration of head and neck squamous cell carcinoma (HNSCC) cells and suppressed the response to cisplatin. (**A**) Collagen significantly increased the growth of the HNSCC cell lines, SCC040, SCC154, VU040T and VU147T. (**B**) Pre-treatment of cells with collagen significantly enhanced the migration of cells through fibronectin-coated membranes. (**C**) Cells pre-treated with collagen were significantly less sensitive to cisplatin, as determined by Annexin V-FITC/Propidium Iodide staining following treatment with 50 μM cisplatin for 72 hours. Results shown are mean +/− standard deviation values of triplicates. *, **, ***, **** denote *p* < 0.05, *p* < 0.01, *p* < 0.001 and *p* < 0.0001 respectively.

Having shown that HNSCCs exist in a collagen-rich environment, we examined the effects of exogenous type I collagen, which is frequently used as an activator of DDR1, on the behaviour of HNSCC cell lines (SCC040, SCC154, VU040T and VU147T) using assays of cell growth, migration and response to chemotherapy in vitro. To examine the effect of collagen on proliferation, cells were grown in the absence and presence of collagen and cell growth measured by cell counting. The exogenous addition of collagen significantly promoted the growth of all four cell lines compared to cells grown in the absence of collagen (Figure 5A).

Transwell assays were used to investigate the effects of collagen on the migration of HNSCC cells. The migration of the cells pre-treated with collagen was significantly higher than cells pre-treated with gelatin or untreated controls (*p* < 0.001; Figure 5B). 

Next, we examined whether collagen affected the response of VU040T and VU147T cells to cisplatin. Cells were pre-treated with or without collagen or gelatin for 24 hours prior to treatment with cisplatin. Apoptosis was measured by flow cytometric analysis of annexin V staining. Pre-treatment of both VU040T and VU147T cells with collagen significantly suppressed apoptosis following treatment with cisplatin compared to the controls (*p* < 0.01; Figure 5C).

### 2.4. The Effects of Collagen Are Mediated through DDR1 in HNSCC Cells

**Figure 6:**
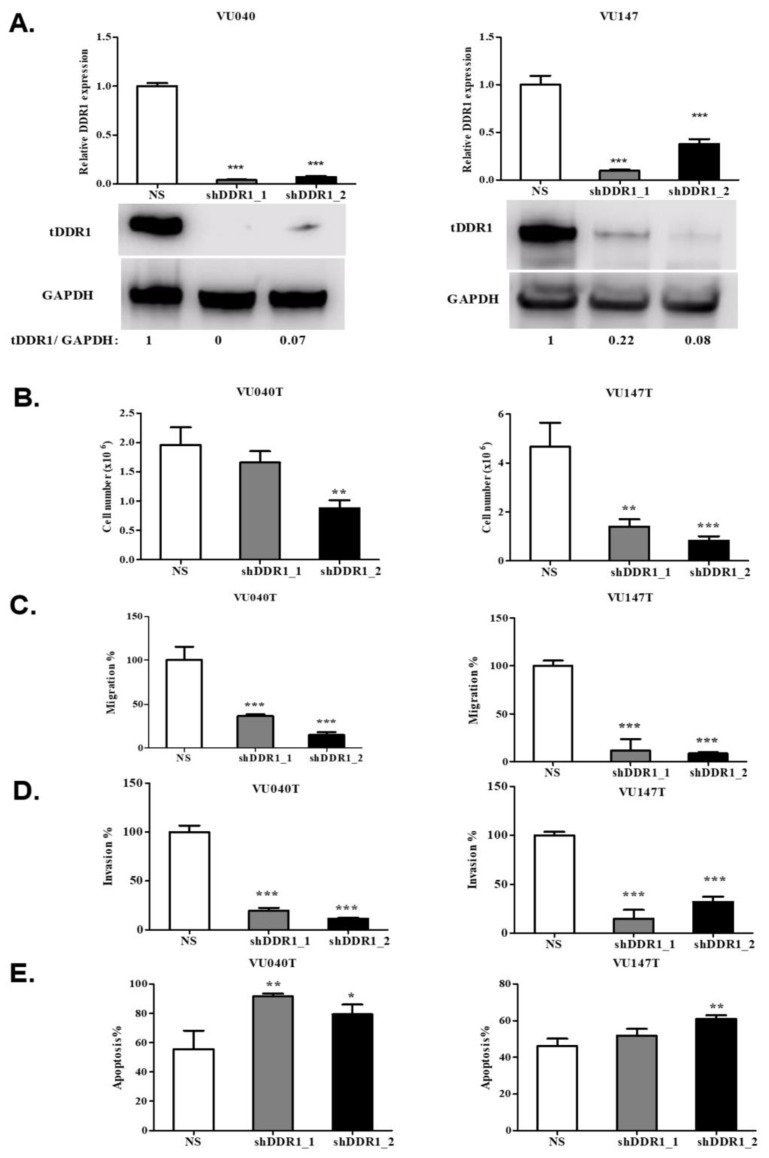
The effects of collagen are mediated by discoidin domain receptor 1 (DDR1). (**A**) Knockdown of DDR1 following stable transduction of VU040 and VU147T cells with two independent short hairpin RNAs. RT-qPCR showed a marked reduction in DDR1 mRNA (top panel) and total DDR1 protein levels (bottom panel). The expression of DDR1 mRNA in NS (control) cells was normalized to 1. (**B**) Following DDR1 knockdown, VU040 and VU147T cells grew slower compared to NS controls in the presence of collagen. (**C**) Knockdown of DDR1 inhibited the migration of VU040T and VU147T cells pre-treated with collagen in Transwell assays. (**D**) Knockdown of DDR1 inhibited the invasion of VU040T and VU147T cells through matrigel-coated filters in Transwell assays. (**E**) The protective effects of collagen on cisplatin-induced apoptosis were reversed following DDR1 knockdown. Results shown are mean +/- standard deviation values of triplicates. *, **, *** denotes *p* < 0.05, *p* < 0.01 and *p* < 0.001 and *p* < 0.0001 respectively.

We next investigated the role of DDR1 in mediating the biological effects of collagen on HNSCC cells. To do this, we generated cells that stably expressed short hairpin RNA (shRNA) targeting DDR1. We transduced SCC040, SCC154, VU040T and VU147T cells with two different shRNAs or with a non-targeting shRNA (NS) as a control. Reduced expression of both DDR1 mRNA and protein was confirmed by RT-qPCR and Western blot analyses, respectively (Figure 6A and Figure S6). 

Knockdown of DDR1 significantly inhibited the growth of VU040T and VU147T cells grown in the presence of collagen (Figure 6B). Transwell assays were performed to determine the migratory and invasive capabilities of VU040T and VU147T cells following DDR1 knockdown. DDR1 knockdown significantly suppressed collagen-stimulated migration (*p* < 0.0001; Figure 6C) and significantly inhibited invasion through matrigel-coated membranes (*p* < 0.0001; Figure 6D) in both cell lines. Finally, we examined whether the effects of collagen on cisplatin sensitivity were mediated by DDR1. Collagen pre-treatment significantly inhibited the cytotoxicity of cisplatin of NS control cells (*p* < 0.001) and these effects were abolished in cells following DDR1 knockdown (*p* < 0.01; Figure 6E). DDR1 knockdown resulted in similar effects in the absence of exogenous collagen, although these effects were less marked, presumably due the effects of endogenous collagen production and autocrine signalling (Figure S6). Taken together, these results demonstrate that the tumour-promoting effects of collagen are mediated via DDR1. 

## 3. Discussion

It is now recognised that proteins secreted from both carcinoma cells and the non-malignant cells within the tumour microenvironment influence epithelial tumour development and progression. Here, we showed that the expression of collagen is higher in HNSCC-derived CAFs than in normal fibroblasts, although there was considerable heterogeneity in the expression of the individual collagen subtypes between CAF strains. Our data support those of Lim and colleagues [12] who showed that the expression of COL8A1 and COL11A1 were elevated in CAFs with a high percentage of senescent cells compared to those with fewer senescent cells. Similar results were obtained in our study, although we found that COL8A1 levels were also higher in some CAFs with a low percentage of senescent cells. We further showed that COL8A1 and COL11A1 are expressed by some HNSCC cell lines and are expressed by both tumour cells and CAFs in HNSCC tissues. Elevated COL11A1 expression in HNSCCs and CAFs has been reported to enhance tumour cell proliferation, migration and invasion [13] and elevated levels of COL11A1 are associated with the progression of carcinomas of different types [14]. There are only a limited number of studies that have investigated the role of COL8A1 in cancer, but COL8A1 expression has been reported to be correlated with the progression and prognosis of colon cancer [15] and knockdown of COL8A1 reduces invasion in hepatocarcinoma cells [16]. Interestingly, we showed that low expression of COL8A1 in OPSCC tumours and CAFs was significantly associated with low risk of death (i.e., HPV-positive, non-smokers). Taken together, these data indicate that HNSCCs are collagen-rich environments and that collagen is produced by HNSCC cells and CAFs, which is likely to be relevant clinically. The significance of collagen in HNSCCs is further supported by reports describing collagen fibre organisation and increased expression of COL3A1 in HNSCCs are associated with poor prognosis [17,18]. 

To investigate possible mechanisms by which collagen could influence the pathogenesis of HNSCC, we examined the expression of DDR1, a receptor tyrosine kinase that is specifically activated by collagen. Our results showed that DDR1 mRNA and protein are over-expressed in both HPV-positive and -negative HNSCCs compared to normal control tissues. Furthermore, in the present study, DDR1 expression was detected in more than 90% of HNSCCs and high expression was associated with a high risk of death from OPSCC and a worse survival outcome, although we acknowledge that our patient cohort was small. Our data are supported by previous studies which showed high expression of DDR1 is associated poor patient prognosis in lung [9], pancreatic [19] and gastric [20] cancers. 

To explore the functional relevance of collagen in the pathogenesis of HNSCC, we treated HNSCC cell lines (both HPV-positive and HPV-negative) with collagen and examined the effect on tumour cell behaviour. Collagen stimulated the proliferation and migration of all cell lines examined in vitro, results that are consistent with reports in other tumour types [8]. Knockdown of DDR1 with shRNAs demonstrated that these effects were mediated by this receptor. Collagen activation of DDR1 also attenuated the response of HNSCC cells to cisplatin, a chemotherapeutic drug used frequently to treat patients. This is consistent with previous reports that showed that the inhibition of DDR1 can improve the efficacy of chemotherapy in pancreatic ductal carcinoma [21] and that the ectopic expression of DDR1 significantly increased the survival of lymphoma cells after chemotherapeutic drug treatment [22]. These results are important because the development of cisplatin resistance is a common cause of treatment failure in HNSCC patients, particularly those with advanced disease [23,24]. Our data suggest that the inhibition of DDR1 activation by collagens could be useful as a therapeutic strategy and might enhance the therapeutic response to cisplatin. With regards to the suitability of DDR1 as a therapeutic target, it is important to note that DDR1-null mice are viable, but these animals show glomerular defects and exhibit a high incidence of osteoarthritis [25,26]. The role of DDR1 in normal adult tissues is less well understood, but DDR1 is widely expressed in the epithelial cells of many tissues and transcripts are also detectable at high levels in brain, lung, spleen and placenta [27]. Therefore, short-term use of DDR1 inhibitors or strategies to target DDR1 in tumour cells might be required to limit toxicity. 

## 4. Materials and Methods 

### 4.1. Cell Lines and Tissue Samples

A series of human oral fibroblasts cell lines that included normal human oral fibroblasts (NHOF4, NHOF6, NHOF7) and human HNSCC CAFs (BICR3F, BICR31F, BICR59F, BICR63F, BICR66F, BICR69F, BICR70F, BICR73F, BICR78F) were used. The derivation and culture of these fibroblast strains has been described previously [12,28]. 

Four well-characterized human HNSCC cell lines were used. Of these, two were HPV negative (SCC040, VU040T) and two were HPV type 16 positive (SCC154, VU147T). The culture of these lines has been described previously [29,30].

Formalin-fixed paraffin embedded (FFPE) OPSCC samples (*n* = 56) were obtained from the Institute of Head and Neck Studies and Education (InHANSE), Institute of Cancer and Genomic Sciences, University of Birmingham, United Kingdom. The cases were divided into three groups with regards to the risk of death (i.e., low, moderate and high), taking into consideration the tumour stage, nodal status, pack years of tobacco smoking and HPV status [31]. FFPE OSCC samples (*n* = 44) were obtained from the Malaysian Oral Cancer Database and Tissue Bank System (MOCDTBS) managed by the Oral Cancer Research and Coordinating Centre (OCRCC), University of Malaya. Ethical approval for this study was obtained for the use of OPSCCs (REC reference 10/H1210/9) and OSCCs (REC reference: DF OB1602/0026(U)). Selected socio-demographic and clinico-pathological parameters of the OPSCC and OSCC cases are shown in Tables S3 and S4, respectively.

### 4.2. RNA Sequencing and Bioinformatics

Total RNA was extracted from the fibroblast strains and, after rRNA removal and library preparation, RNA sequencing (HiSeq 2000, Illumina, San Diego, CA, USA) was performed by BGI Tech Solutions (Hong Kong) Co Ltd. Sequence reads were aligned to hg19 reference sequence using subread aligner and mapped sequencing reads were assigned to hg19 refGene genes using featureCounts. RefGene exon coordinates were obtained from the University of California Santa Cruz (UCSC) table browser. Gene symbol and description was obtained from the NCBI gene database. The total number of mapped human sequence reads were used for the calculation of counts per million (CPM) of human genes. Differentially-expressed genes were identified using the voom-limma [32] method with the criteria of *p*-value < 0.05, absolute fold change >1.5 and read count ≥10 in at least two samples. The primary data are available in the Gene expression Omnibus database; accession number GSE135975).

A heatmap of relative collagen gene expression was produced using the gplots library in R. Only collagen genes for which the average CPM was at least 1.0 across all samples were used. CPM values for each relevant gene were first centred (at zero) and scaled (and capped at +/− 3). The Euclidean distance measure was then used in conjunction with the Ward.D2 clustering algorithm. For comparing average expression of the same genes across all samples, first the average CPM across the three NHOF samples was calculated for each relevant gene. Then, for each sample the ratio of that sample’s CPM to the average NHOF CPM was calculated for each relevant gene, and the average of these values across all genes was taken. Finally, these values were converted to log(base2) scale.

For gene set enrichment analysis, GSEAv3.0 was used [33,34] in conjunction with the MSigDB database of gene ontology terms (input table = “c5.bp.v6.2.symbols.gmt”) [35]. Genes were pre-ranked according to sign of fold change divided by p-value taken from the differential expression analysis. Default parameters were used except for “Enrichment statistic” which was set as “classic”.

### 4.3. Analysis of DDR1 Expression in the Cancer Genome Atlas HNSCC Data Set

Data from TCGA were downloaded using the “Data matrix” option within the TCGA data portal [36]. Level 3 RNA sequencing data, based on the Illumina HiSeq 2000 RNA Sequencing platform (Version 2; Illumina, San Diego, CA, USA), were downloaded along with all available clinical data in the “Biotab” format. Tumour samples were categorized as either “HPV-negative” or “HPV-positive” according to the data item “HPV status” provided in TCGA’s clinical data. In total there were 400 HPV-negative tumour samples (282 male, 118 female) and 94 HPV-positive samples (84 male, 10 female) for which RNAseq data were also available. In addition, RNAseq data were available for 44 “normal” samples, 37 of which were matched to HPV-negative tumour samples and 6 to HPV-positive tumour samples.

The RNAseq files labelled “rsem.genes.results” contained unnormalised read counts for over 20,000 genes. Read counts were normalised between samples and converted to CPM reads for each gene using the edgeR package [37] in R [38]. Differential expression analysis was also performed using edgeR. A significance level of 5% was used in all statistical tests. 

### 4.4. Western Blotting 

Cells were lysed in ice-cold NP40 lysis buffer (150mM NaCl, 1% IGEPAL® CA-630, 50mM Tris-HCl (pH 8.0)) containing protease inhibitors (cocktail set III; Calbiochem, Merck Millipore, Darmstadt, Germany) and phosphatase inhibitors (Halt phosphatase inhibitor cocktail; Thermo Scientific, Waltham, USA). The primary antibodies used in this study were anti-DDR1 (DIG6 XP; 1:1000; Cell Signaling Technology, Danvers, MA, USA) and anti-GAPDH (ab371681; 1:1000; Abcam, Cambridge, UK). Bound antibodies were detected with peroxidase conjugated secondary antibodies and enhanced chemiluminescence reagents (Advansta, Menlo Park, CA, USA). Signal intensities were measured using ImageJ software.

### 4.5. Immunohistochemistry

Immunohistochemistry (IHC) was performed using standard protocols. For chromogenic detection of DDR1, DAKO REAL EnVision Detection was used (Dako, Agilent Technologies, Santa Clara, CA, USA). Multiplex immunofluorescence (IF) was performed using Opal 7-Plex Kit (NEL791001KT; Perkin-Elmer, Waltham, MA, USA). For antibody stripping between each step, slides were microwaved in pH6 citrate buffer, with controls omitting individual antibody steps to ensure adequate stripping. The primary antibodies used were anti-DDR1 (DIG6 XP, 1:1000; Cell Signaling Technology, Danvers, MA, USA), anti-pan-cytokeratin AE1/AE3 (CK AE1/ AE3, 1:1000; Dako, Agilent Technologies, Santa Clara, CA USA), anti-α-SMA (1:1000; Dako, Agilent Technologies, Santa Clara, CA, USA), anti-collagen VIII alpha 1 (COL8A1, 1:200; Sigma-Aldrich, St Louis, MO, USA) and anti-collagen XI alpha 1 (COL11A1, 1:200; Sigma-Aldrich, St Louis, MO, USA). Validation of the specificity of the DDR1 antibody is shown in Figure S7. 

Multiplex fluorescent staining on tissue sections was quantitatively evaluated using Metamorph Microscopy Automation and Image Analysis software (Molecular Device LLC, San Jose, CA, USA). The area and co-localised integrated intensity of dual fluorescent probes were measured quantitatively for the degree of overlap. The total staining intensity, average intensity and maximum and minimum intensities were obtained by region statistics and region measurements. Means from random five fields were used in analyses for each case. 

### 4.6. Cell Proliferation Assays

1 × 10^5^ cells per dish were seeded into 60 mm dishes in triplicate. Media were changed every 4 days over a period of 14 days. To examine the effect of collagen, the cells were treated with 100 µg/mL of collagen (Merck Millipore, Darmstadt, Germany) and cell numbers determined at 4-day intervals by trypsinisation and cell counting using a Luna Automated Cell Counter (Logos Biosystems, Gyeonggi-do, Korea). 

### 4.7. Transwell Migration and Invasion Assays

Cells were grown in 75 cm^2^ flasks to reach 80% confluence and incubated with or without collagen (Merck Millipore, Darmstadt, Germany) or gelatin (Sigma-Aldrich, St Louis, MO, USA) for 24 hours. Prior to use, the cells were treated with a final concentration of 10 µg/mL mitomycin C (Merck Millipore, Darmstadt, Germany) at 37 °C for 2 hours to negate the effects of cell proliferation. Polycarbonate inserts of 8 µm pore size (Transwell, Corning, Cambridge, MA, USA) in 24-well plates were coated with 200 µL of 10 µg/mL fibronectin (Thermo Scientific, Waltham, USA) at 37 °C for 2 hours prior to use. 1 × 10^6^ cells in 200 µL medium were seeded into the upper chambers and 500 µL of medium enriched with 20% FBS was loaded into the lower chamber. Migrated cells were stained with 0.1% crystal violet and counted in five random fields. For invasion assays, pre-coated invasion chambers (Corning Matrigel Invasion Chamber, 8 µm pore size, Corning, Cambridge, MA, USA) were used and cells allowed to invade for 48 h.

### 4.8. Annexin V-FITC/ Propidium Iodide (PI) Apoptosis Assays

Cells treated with Cisplatin (Tocris Bioscience, Minneapolis, MN, USA) with or without collagen (Merck Millipore, Darmstadt, Germany) pre-treatment were collected using Accutase Cell Detachment Reagent (BD Biosciences, San Jose, CA, USA) together with the dead cells in the medium. Apoptosis was examined using an Annexin V: FITC apoptosis detection kit (BD Biosciences, San Jose, CA, USA).The cells were stained with Annexin V-FITC and Propidium Iodide (PI) and analysed using a BD FACS Canto II Flowcytometer (BD Biosciences, San Jose, CA, USA). Apoptosis was quantified by expressing the total number of cells in early (Q2) and late (Q4) apoptosis as a percentage of the total number of cells (Q1–Q4).

### 4.9. Reverse Transcription-Quantitative PCR (RT-qPCR)

Total RNA was extracted using an RNeasy Mini Kit (Qiagen, Hilden, Germany) and subjected to reverse transcription using High-Capacity cDNA Reverse Transcription kit (Applied Biosystems, Foster City, CA, USA). Q-PCR was performed in triplicate using the ABI Prism 7000 Sequence Detection System and TaqMan Gene Expression Assays (Applied Biosystems, Foster City, CA, USA; COLVIIIa1; Hs00156669_m1: COLXIa1; Hs01097664_m1: DDR1; Hs01058430_m1: GAPDH; 4326317E). GAPDH was amplified in the same reaction to serve as an internal control for normalization. Fold changes in gene expression were measured using the comparative threshold cycle method (ΔΔCt).

### 4.10. Knockdown of DDR1

Two DDR1 shRNA lentiviral plasmids (pLKO.1/shDDR1_10084, pLKO.1/shDDR_1121293) and the non-targeting (control) shRNA (pLKO.1/NS) were obtained from Sigma Aldrich (Sigma-Aldrich, St Louis, MO, USA). Briefly, 293T cells were transfected with the lentiviral construct using polyethylenimine together with the packaging plasmid (psPAX2) and envelope plasmid (pMDG2). After 48 hours the cells were incubated with viral supernatants containing polybrene (8 µg/mL) for 18 hours. The virus-containing media were then removed and the cells cultured in Roswell Park Memorial Institute (RPMI) 1640/10% fetal bovine serum for an additional 48 hours before selection with 2 μg/mL puromycin.

### 4.11. Statistical Analyses

Apart from the bioinformatics analyses described separately, all the statistical analyses were carried out using GraphPad PRISM 5.0 software (GraphPad Software, San Diego, CA, USA) and SPSS version 22. Statistical analysis for the IHC was undertaken by categorizing the means of final immunoreactive scores into 2 groups. The cut-off points for these groupings were derived based on the optimal sensitivity and specificity obtained from receiver operating characteristic (ROC) curve analyses. The associations between the expression of COL8A1, COL11A1 and DDR1 with selected clinico-pathological parameters were analysed using univariate and multivariate logistic regression. Survival curves were plotted using the Kaplan–Meier analysis to correlate survival with DDR1 expression and the survival probability differences were compared by log-rank tests. Differences between groups were evaluated by ANOVA, followed by the Dunnett’s test for post hoc analysis. p-values less than 0.05 were considered to be statistically significant.

## 5. Conclusions

In summary, we report for the first time that DDR1 is over-expressed in HNSCCs and is likely to be activated in vivo by collagen produced by tumour cells and CAFs to promote tumorigenesis and chemotherapy resistance. Selective inhibitors of DDR1 are being developed and show efficacy in preclinical models of fibrosis and cancer [21,39,40]. Our data suggest that inhibition of collagen-induced DDR1 activity could represent a novel therapeutic strategy for the treatment of HNSCC. 

## Supplementary Materials

The following are available online at https://www.mdpi.com/2072-6694/11/11/1766/s1, Figure S1: Expression of collagen in NHOFs and CAFs, Figure S2: Expression of COL8A1 and COL11A1 in OSCC, Figure S3: Figure 3A full blot, Figure S4: Expression of DDR1 in HNSCC cell lines and non-malignant keratinocytes, Figure S5: Expression of DDR1 in OSCC, Figure S6: Effect of DDR1 knockdown on cell proliferation, migration and response to cisplatin, Figure S7: Validation of the specificity of the antibody against DDR1, Table S1: Association between DDR1 and COL8A1 protein expression with OPSCC risk of death groups, Table S2: Overexpression of DDR1 in HNSCC, Table S3: Socio-demographic and clinico-pathological characteristics of OPSCC cases, and Table S4: Socio-demographic and clinico-pathological characteristics of OSCC cases.
