# Supplementary material for: Collagen Induces a More Proliferative, Migratory and Chemoresistant Phenotype in Head and Neck Cancer via DDR1
Source: Cancers (Basel). 2019 Nov 9;11(11):1766. doi: 10.3390/cancers11111766 (PMC6896141; doi:10.3390/cancers11111766)
Supplement: Supplementary file 1 [file cancers-11-01766-s001.zip › Supplementary files Word/Supplementary figures.docx]

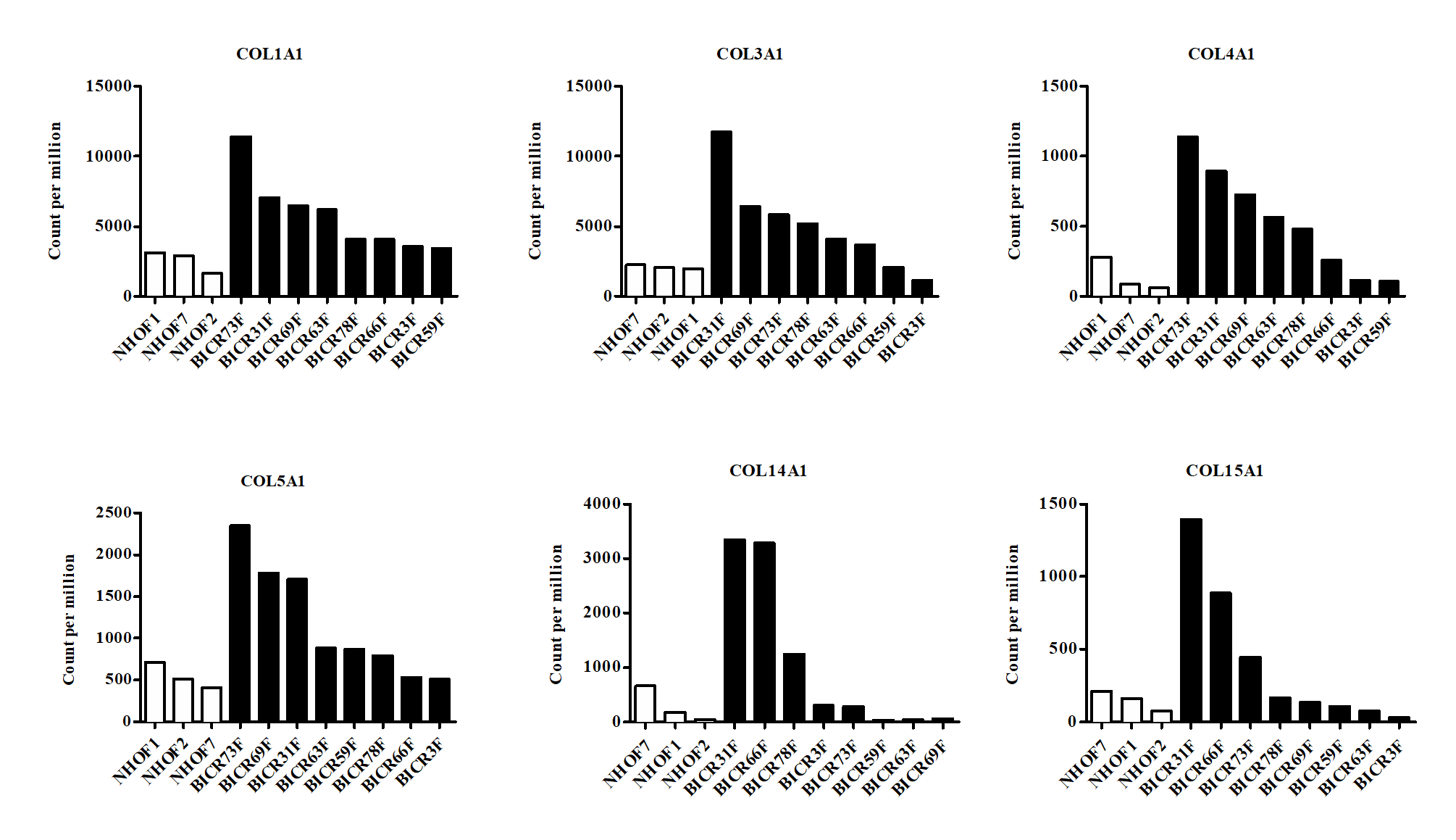


**Figure S1: Expression of collagen subtypes in NHOFs and CAFs.** RNAseq analysis showed overexpression of collagen subtypes in CAFs.


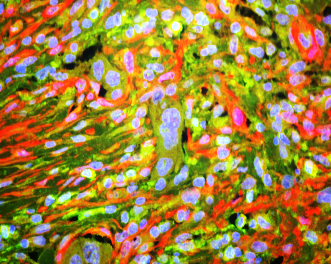

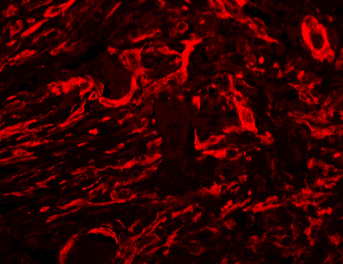

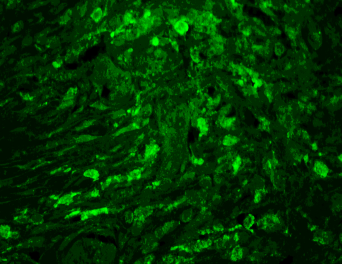

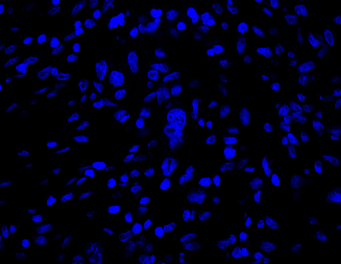

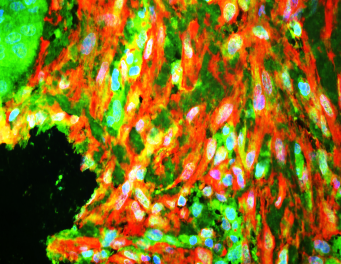

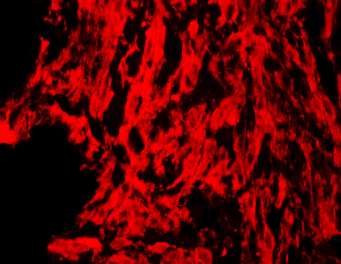

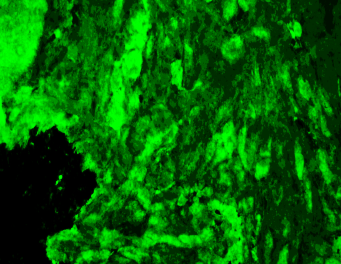

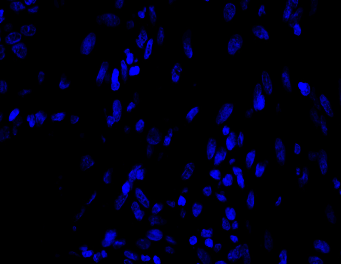


**α-SMA**

**COL8A1**

**Dapi**

**Merged**

**α-SMA**

**COL11A1**

**Dapi**

**Merged**

**Figure S2: Expression of Col 8A1 and Col 11A1 in OSCC.** Expression of COL8A1 (top panel) and COL11A1 (lower panel) in OSCC tissues. Tissues were multiplex-stained with COL8a1 or COL11A1 (fluorescein, green), together with α-SMA (Cy3, red) antibodies and DAPI (blue). The expression of COL8A1 and COL11A1 was detected in OPSCCs and their associated CAFs. Representative images are shown and were captured using Metamorph Pathology Imaging System (Nikon, x60).


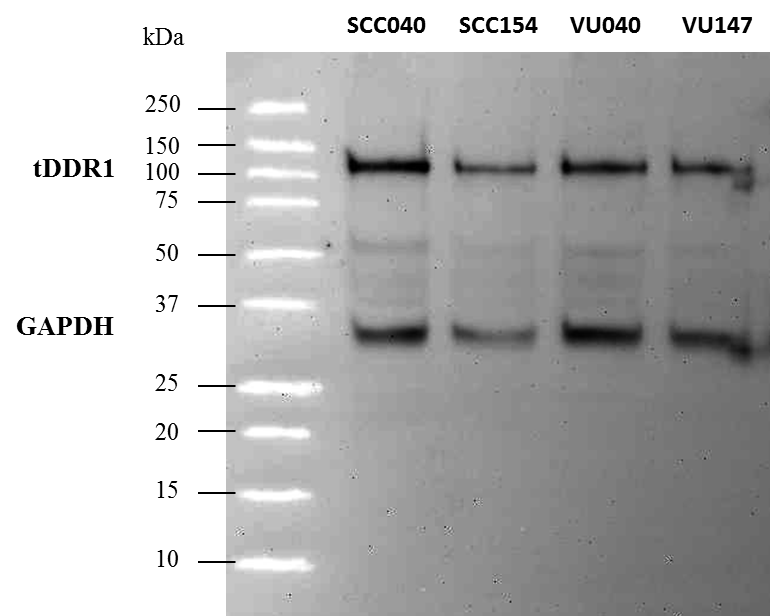


**Figure S3: Full blot shown in Figure 3A**


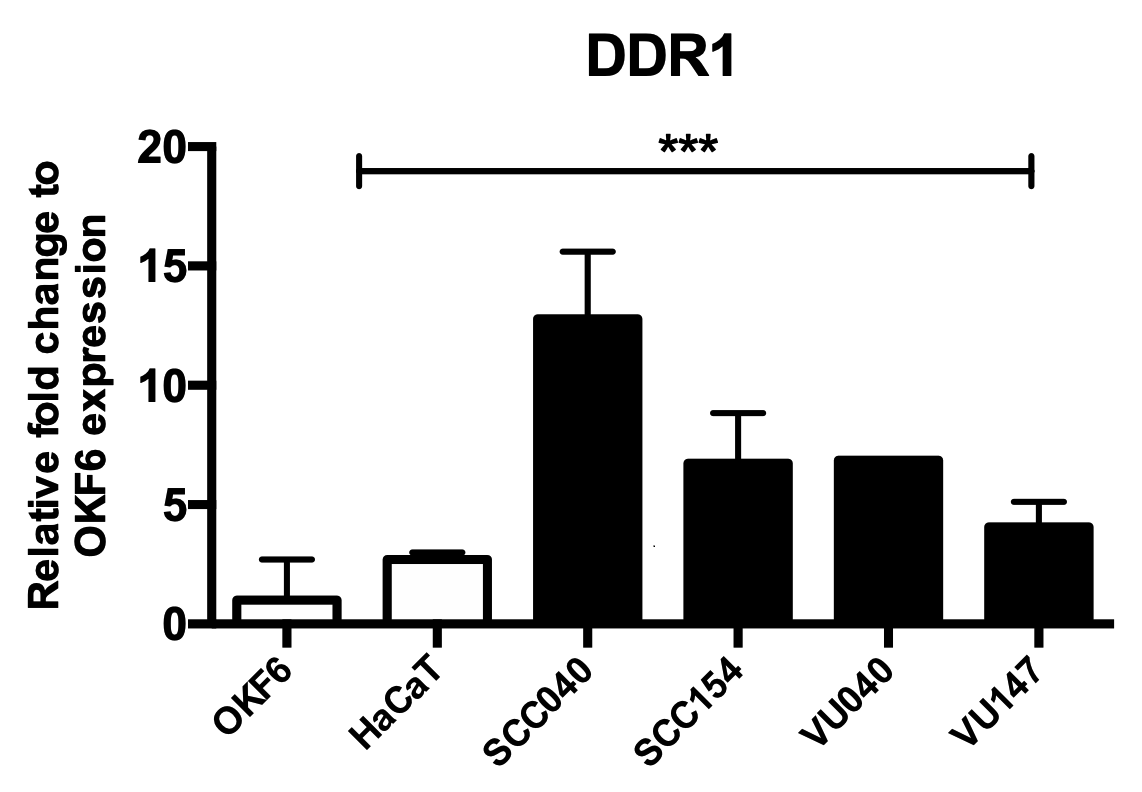


**Figure S4: Expression of DDR1 in HNSCC cell lines and non-malignant keratinocytes.** QPCR analysis of DDR1 expression in HNSCC cell lines and immortalized normal human oral keratinocytes (OKF6) and non-malignant epidermal keratinocytes (HaCaT). The data indicate that DDR1 expression is higher in SCC cell lines. *** p<0.001, Welch test for unequal variance.


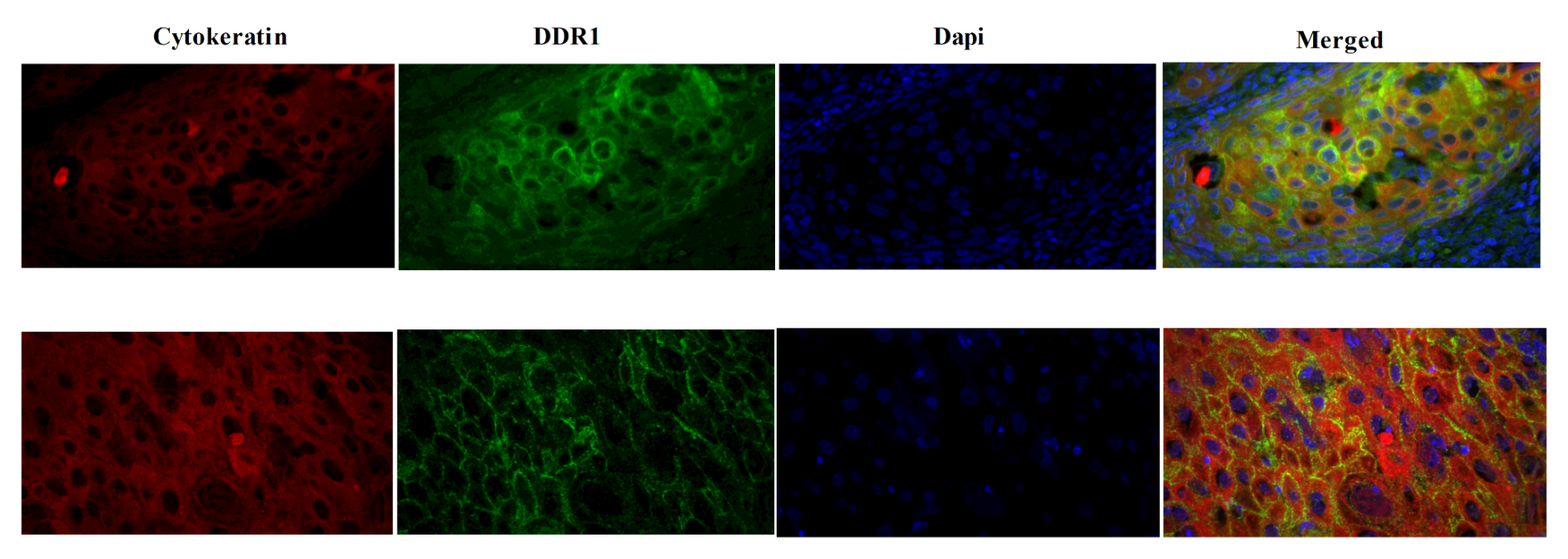


**Figure S5: Expression of DDR1 in OSCC.** Tissues were multiplex-stained with CK AE1/ AE3 (Cy3, red) and DDR1 (fluorescein, green) antibodies, plus DAPI (blue) nuclear counterstain. DDR1 expression in OSCCs was cytoplasmic and membranous (top panel) or predominantly membranous (bottom panel). Representative images are shown and were captured using Metamorph Pathology Imaging System (Nikon, x60).


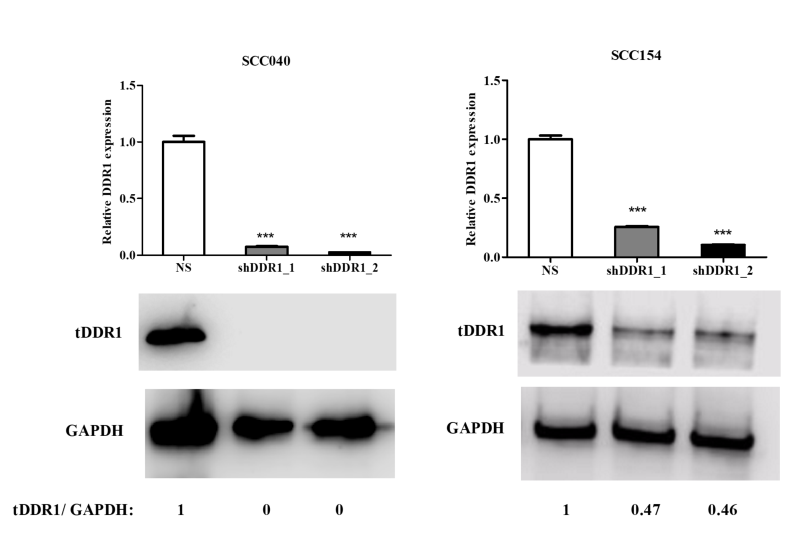

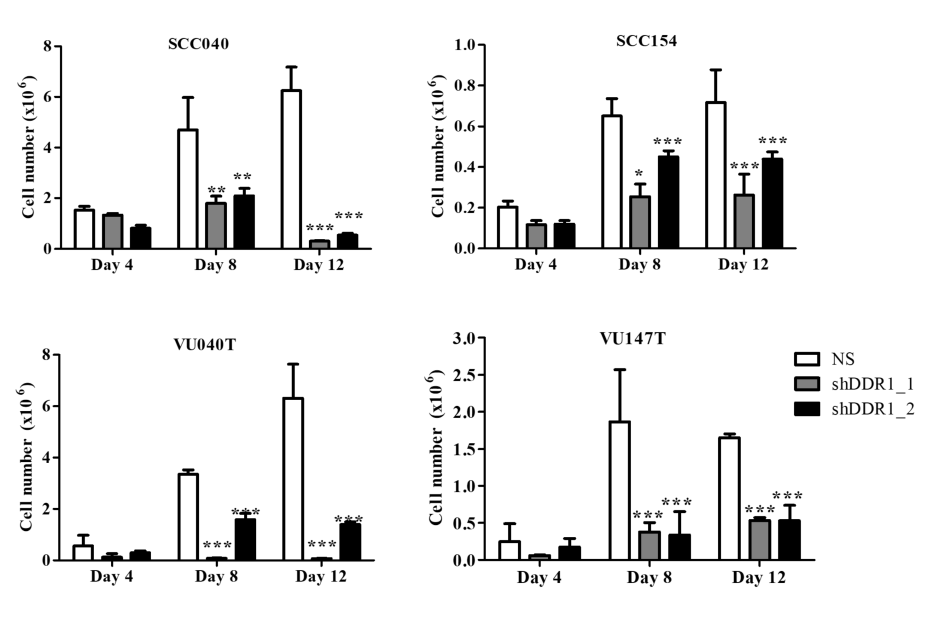

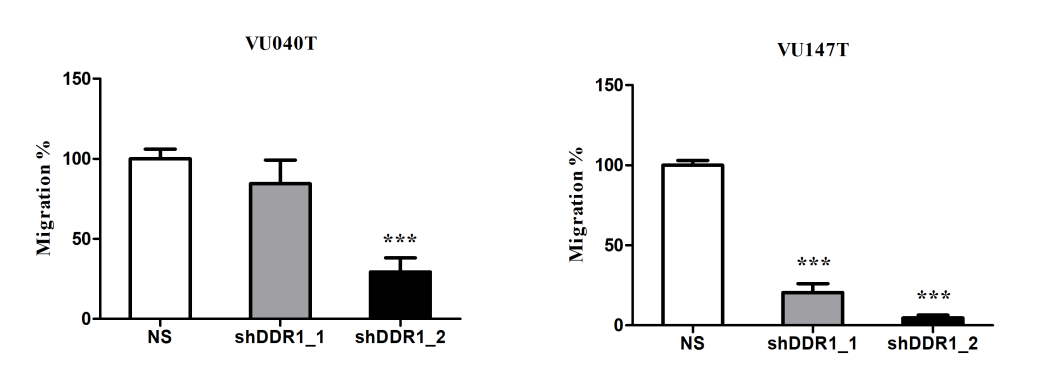

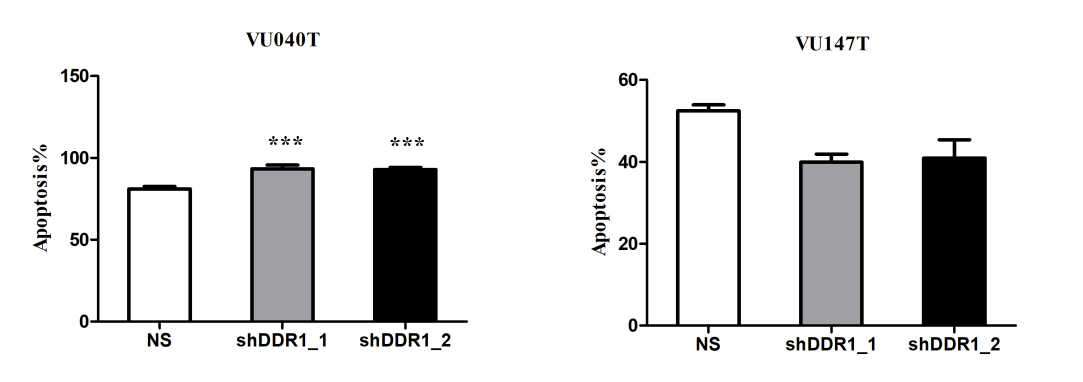


**A**

**C**

**D**

**B**

**Figure S6: Effect of DDR1 knockdown on cell proliferation, migration and response to cisplatin.** A. Knockdown of DDR1 in SCC40 and SCC154. B. DDR1 knockdown inhibited the growth of all cell lines cultured in the absence of collagen, presumably by abrogating the growth promoting effects of endogenous collagen. C. Knockdown of DDR1 inhibited the migration of VU040T and VU147T cells in Transwell assays. D. Cisplatin-induced apoptosis following DDR1 knockdown. Results shown are mean +/- SD values of triplicates. *, **, *** denotes p<0.05, p<0.01 and p<0.001, respectively.


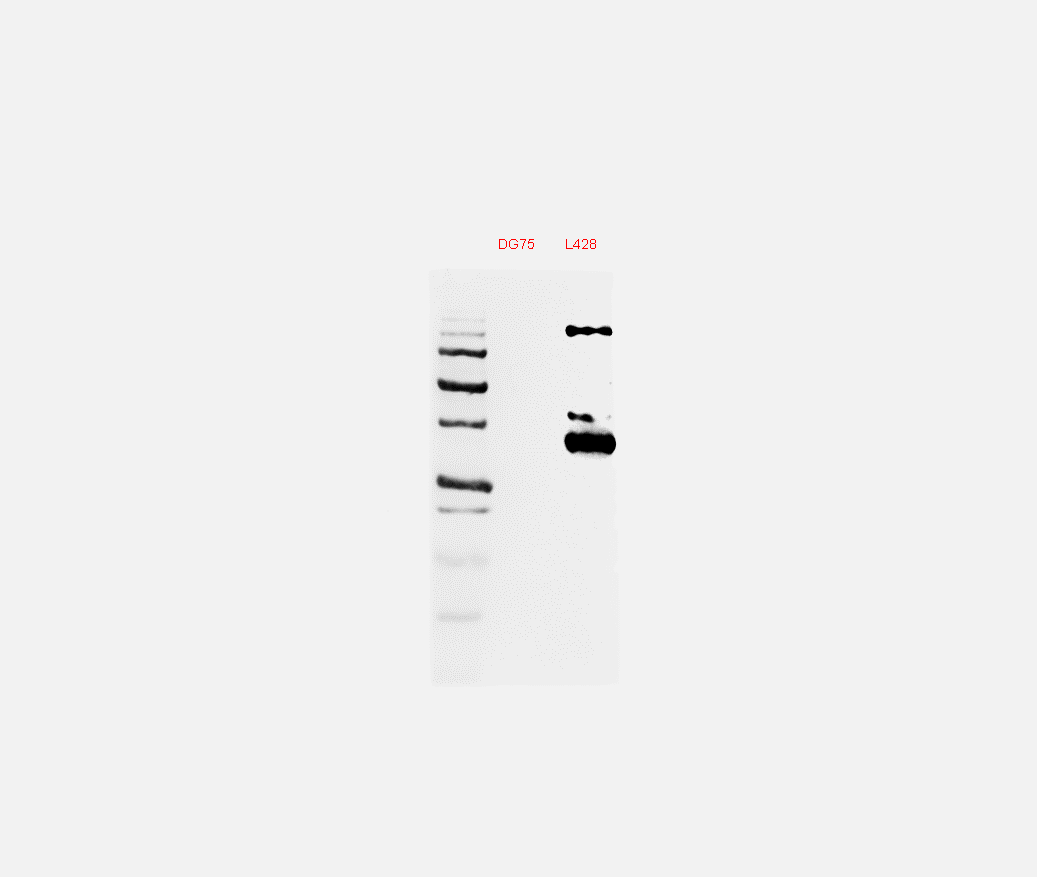

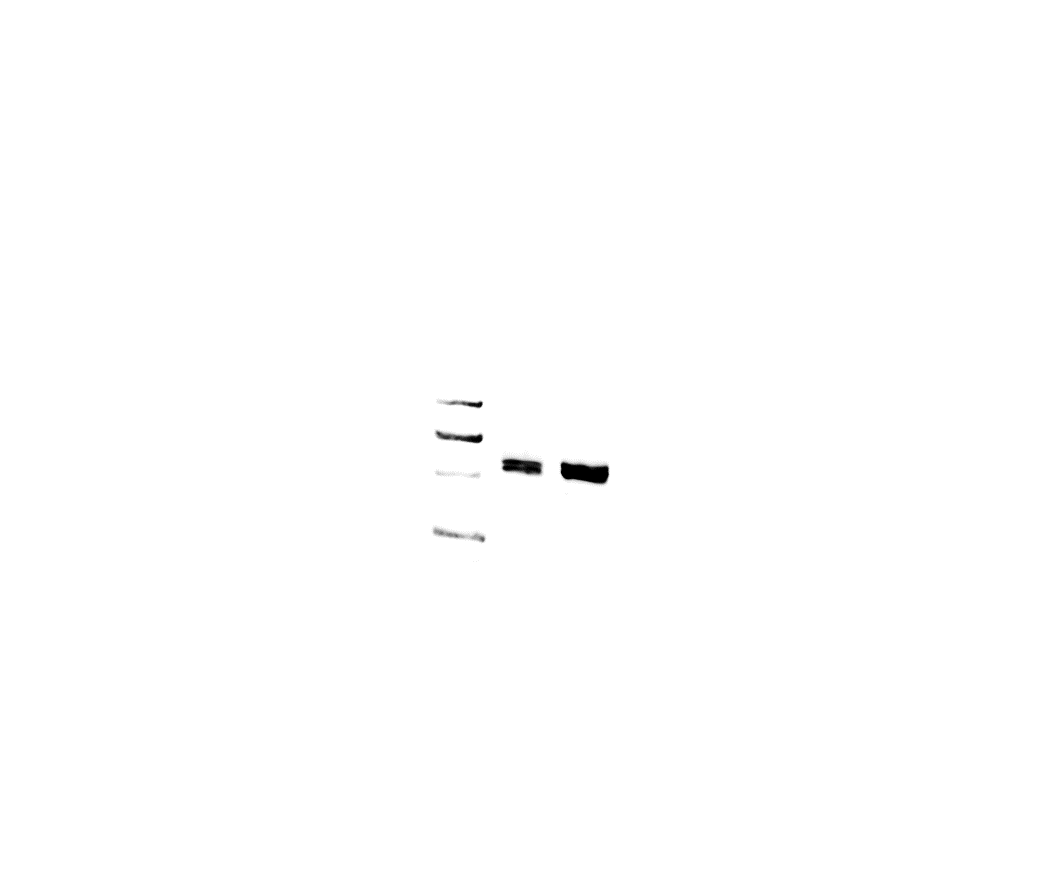


DG75

L428

DDR1 (125kDa)

MCM7 (80kDa)

A.

B.

DG75 + DDR1 plasmid

DG75 + empty vector


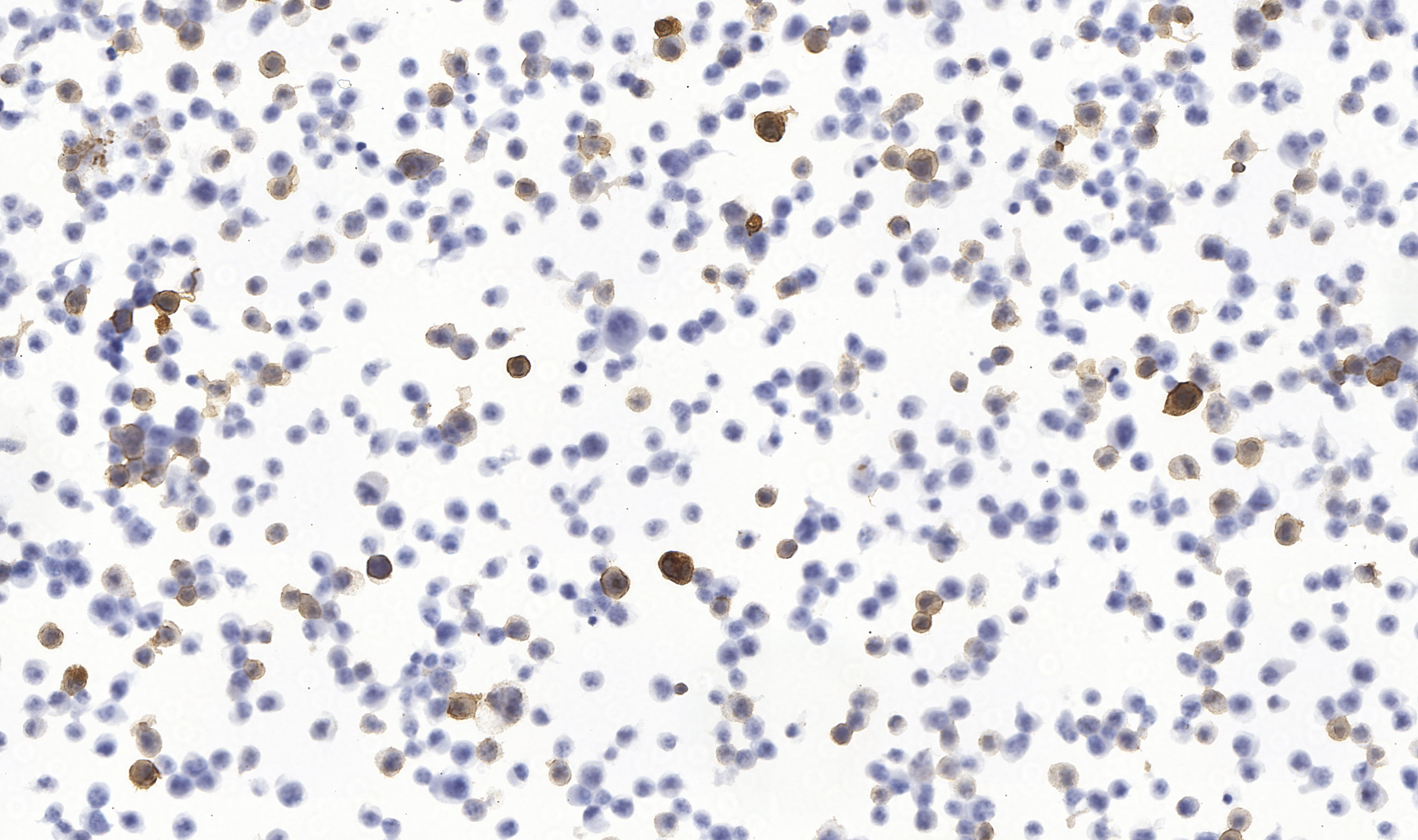

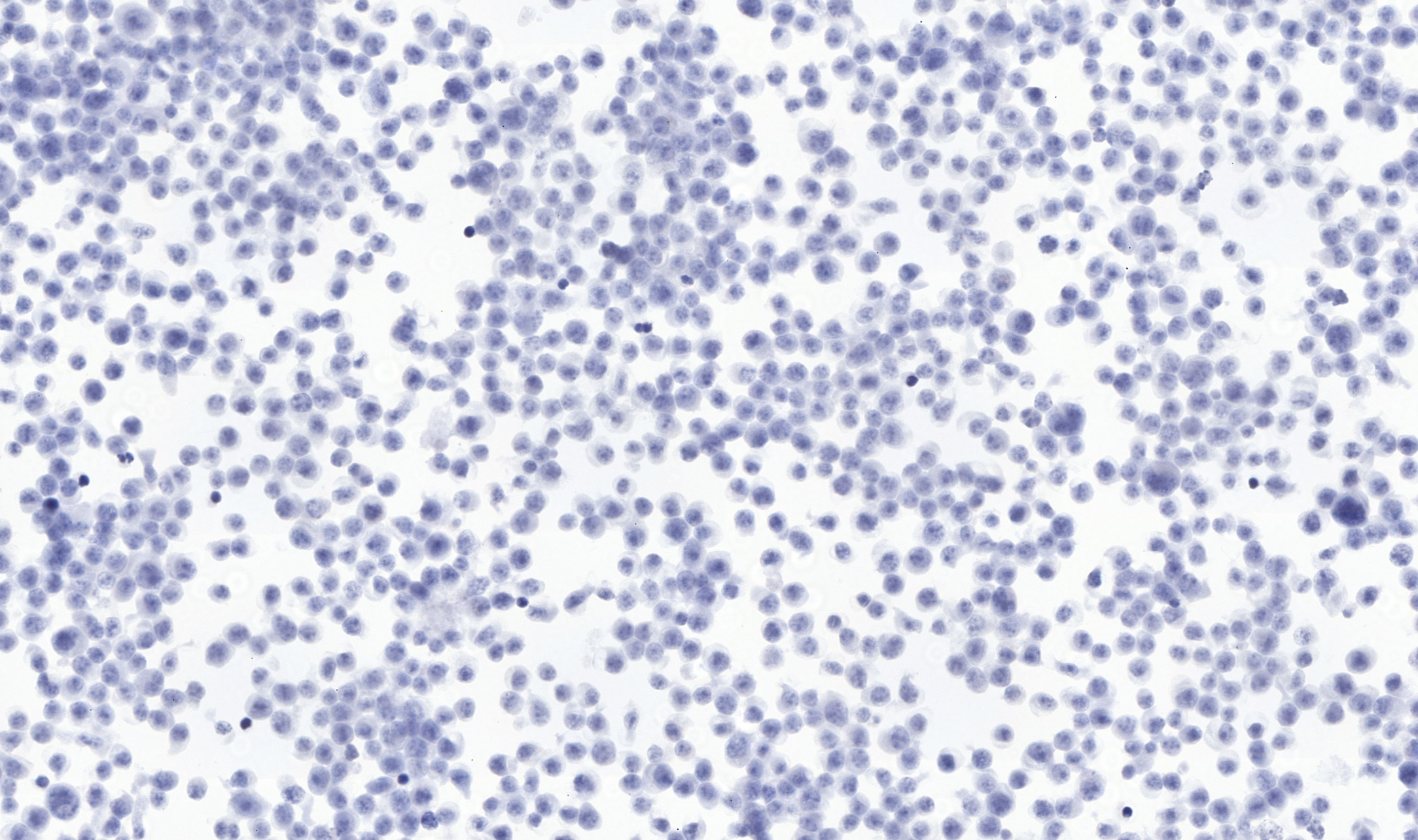


**Figure S7: Validation of the specificity of the antibody against DDR1.** A. Western blot analysis of DDR1 expression in cells negative (DG75, Burkitt lymphoma cells) and positive (L428, Hodgkin lymphoma cells) for DDR1 protein expression. B. The antibody was further validated by immunostaining using DG75 cells transfected with empty vector or a DDR1 plasmid.
