# Supplementary material for: Collagen Induces a More Proliferative, Migratory and Chemoresistant Phenotype in Head and Neck Cancer via DDR1
Source: Cancers (Basel). 2019 Nov 9;11(11):1766. doi: 10.3390/cancers11111766 (PMC6896141; doi:10.3390/cancers11111766)
Supplement: Supplementary file 1 [file cancers-11-01766-s001.zip › Supplementary files Word/Supplementary tables.docx]

**Table S1: Association between DDR1 and COL8A1 protein expression with OPSCC risk of death groups**

| **Categories** | | **DDR1 Expression (% of total)** | | | **Univariate Logistic Regression** | | | | |
| --- | --- | --- | --- | --- | --- | --- | --- | --- | --- |
|  |  | Low | High | | Odd ratio | 95% CI | | p-value | |
| Risk Group | Low | 9.1 | 0.0 | | 4.667 | (1.11,19.65) | | *0.036 | |
|  | Moderate | 21.8 | 25.5 | | 0.972 | (0.25,3.85) | | 0.968 | |
|  | High | 18.2 | 12.7 | | 1.000 | - | | - | |
|  | NA | 1.8 | 10.9 | | 0.000 | (0.00,-) | | 1 | |
| **Categories** | | **COL8A1 expression in tumour cells (% of total)** | | | **Univariate Logistic Regression** | | | | |
|  |  | Low | | High | Odd ratio | | 95% CI | | p-value |
| Risk Group | Low | 29.5 | | 6.8 | 0.069 | | (0.01,0.42) | | *0.004 |
|  | Moderate | 15.9 | | 15.9 | 0.300 | | (0.06,1.58) | | 0.156 |
|  | High | 6.8 | | 22.7 | 1.000 | | - | | - |
|  | NA | 0.0 | | 2.3 | 0.000 | | (0.00,-) | | 1.000 |
| **Categories** | | **COL8A1 expression in tumour cells (% of total)** | | | **Univariate Logistic Regression** | | | | |
|  |  | Low | | High | Odd ratio | | 95% CI | | p-value |
| Risk Group | Low | 25.0 | | 11.4 | 0.202 | | (0.04,0.98) | | *0.048 |
|  | Moderate | 13.6 | | 18.2 | 0.593 | | (0.12,2.89) | | 0.517 |
|  | High | 9.1 | | 20.5 | 1.000 | | - | | - |
|  | NA | 2.3 | | 0.0 | 0.000 | | (0.00,-) | | 1.000 |

Cases were divided into three groups with regards to the risk of death (i.e. low, moderate and high), taking into consideration the tumour stage, nodal status, pack years of tobacco smoking and HPV status [26].

**Table S2. Overexpression of DDR1 in HNSCC**

| **Site** | **Epithelium intensity** | **Tumour intensity** |
| --- | --- | --- |
| Lip mucosa | 1 | N/A |
| Buccal mucosa | 1 | N/A |
| Tongue dorsum | 1 | N/A |
| Palate | 1 | N/A |
| Pharyngeal tonsil | 1 | N/A |
| OPSCC 1 | 1 | 3 |
| OPSCC 2 | 1 | 2 |
| OPSCC 3 | 2 | 3 |
| OPSCC 4 | 1 | 2 |
| OPSCC 5 | 2 | 2 |
| OPSCC 6 | 1 | 3 |
| OSCC 1 | 1 | 1 |
| OSCC 2 | 1 | 1 |
| OSCC 3 | 1 | 2 |
| OSCC 4 | 1 | 2 |
| OSCC 5 | 2 | 2 |
| OSCC 6 | 1 | 2 |

DDR1 staining intensity was higher in the squamous cell carcinoma compared to the adjacent normal epithelium. Weak cytoplasmic (+/- membrane staining) was observed in the squamous epithelium of normal oral tissues. Staining intensity was scored using an arbitrary scale: 1, weak, 2, moderate, and 3, strong staining. N/A, not applicable.

**Table S3: Socio-demographic and clinico-pathological characteristics of OPSCC cases**

| **Characteristic** | | **N=55 (%)** | **Characteristic** | | **N=55 (%)** |
| --- | --- | --- | --- | --- | --- |
| **Smoking** | Never | 19 (34.5) | **Gender** | Male | 37 (67.3) |
|  | Past | 8 (14.5) |  | Female | 18 (32.7) |
|  | Current | 27 (49.1) | **T category** | T0 | 1 (1.8) |
|  | NA | 1 (1.8) |  | T1 | 33 (60.0) |
| **HPV DNA**  **ISH** | Negative | 38 (69.1) |  | T2 | 18 (32.7) |
|  | Positive | 16 (29.1) |  | NA | 3 (5.5) |
|  | No record | 1 (1.8) | **N category** | N1 | 30 (54.5) |
| **p16 IHC** | Negative | 24 (43.6) |  | N2 | 22 (40.0) |
|  | Positive | 30 (54.5) |  | NA | 3 (5.5) |
|  | NA | 1 (1.8) | **Risk of death group** | Low | 19 (34.5) |
| **Outcome** | Alive | 20 (36.4) |  | Moderate | 19 (34.5) |
|  | Deceased | 32 (58.2) |  | High | 16 (29.1) |
|  | NA | 3 (5.5) |  | NA | 1 (1.8) |

**Table S4: Socio-demographic and clinico-pathological characteristics of OSCC cases**

| **Parameters** | **Characteristic** | **n=44 (%)** | **Parameters** | **Characteristic** | **n=44 (%)** |
| --- | --- | --- | --- | --- | --- |
| Smoking | No | 29 (65.9) | Gender | Male | 13 (29.5) |
|  | Yes | 3 (6.8) |  | Female | 31 (70.5) |
|  | No record | 12 (27.3) | Grade | Well | 11 (25.0) |
| Betel chewing status | No | 15 (34.1) |  | Moderate | 27 (61.4) |
|  | Yes | 20 (45.5) |  | Moderate to poor | 1 (2.3) |
|  | No record | 9 (20.5) |  | Poor | 3 (6.8) |
| Alcohol | No | 30 (68.2) |  | No record | 2 (4.5) |
|  | Yes | 2 (4.5) | Stage | I | 2 (4.5) |
|  | No record | 12 (27.3) |  | II | 11 (25.0) |
| Survival | Alive | 8 (18.2) |  | III | 0 (0.0) |
|  | Deceased | 15 (34.1) |  | IV | 26 (59.1) |
|  | Not follow up | 2 (4.5) |  | V | 0 (0.0) |
|  | No record | 19 (43.2) |  | No record | 5 (11.4) |
